# New insights into the regulation by RUNX1 and GFI1(s) proteins of the endothelial to hematopoietic transition generating primordial hematopoietic cells

**DOI:** 10.1080/15384101.2016.1203491

**Published:** 2016-07-11

**Authors:** Roshana Thambyrajah, Rahima Patel, Milena Mazan, Michael Lie-a-Ling, Andrew Lilly, Alexia Eliades, Sara Menegatti, Eva Garcia-Alegria, Magdalena Florkowska, Kiran Batta, Valerie Kouskoff, Georges Lacaud

**Affiliations:** a CRUK Stem Cell Biology, Cancer Research UK Manchester Institute, Manchester, UK; b CRUK Stem Cell Haematopoiesis, Cancer Research UK Manchester Institute, Manchester, UK

**Keywords:** development, ES cells, EHT (endothelial to hematopoietic transition), GFI1, GFI1B, hematopoiesis, HSCs (Hematopoietic stem cells), hemogenic endothelium, RUNX1, reprogramming

## Abstract

The first hematopoietic cells are generated very early in ontogeny to support the growth of the embryo and to provide the foundation to the adult hematopoietic system. There is a considerable therapeutic interest in understanding how these first blood cells are generated in order to try to reproduce this process *in vitro*. This would allow generating blood products, or hematopoietic cell populations from embryonic stem (ES) cells, induced pluripotent stem cells or through directed reprogramming. Recent studies have clearly established that the first hematopoietic cells originate from a hemogenic endothelium (HE) through an endothelial to hematopoietic transition (EHT). The molecular mechanisms underlining this transition remain largely unknown with the exception that the transcription factor RUNX1 is critical for this process. In this Extra Views report, we discuss our recent studies demonstrating that the transcriptional repressors GFI1 and GFI1B have a critical role in the EHT. We established that these RUNX1 transcriptional targets are actively implicated in the downregulation of the endothelial program and the loss of endothelial identity during the formation of the first blood cells. In addition, our results suggest that GFI1 expression provides an ideal novel marker to identify, isolate and study the HE cell population.

The development of the vertebrate hematopoietic system is characterized by 3 successive waves of blood progenitor generation. The first 2 waves of blood cell emergence take place in the extra-embryonic yolk sac and generate mainly primitive erythrocytes by E7.5 and erythroid-myeloid progenitors (EMPs) by E.8.5.^1-6^ It is only during the third wave of blood development that the first hematopoietic stem cells (HSCs) with multi-lineage and long-term repopulating potential arise in the intra-embryonic aorta-gonad-mesonephros (AGM) region.^7-10^ Seminal experiments have indicated that blood cells initially emerge from endothelial cells (i.e. from a hemogenic endothelium).^11-14^ Recent elegant live imaging studies of the AGM region^15-19^ or using differentiated embryonic stem (ES) cells^20-23^ have provided evidences that endothelial cells directly become blood cells through an endothelial to hematopoiesis transition (EHT). This is consistent with the observations that the first progenitors with HSC activity are detected in the major arteries in the developing embryo^24-26^ and that the intra-aortic hematopoietic clusters (IAHC) generated through the EHT in the ventral wall of the dorsal aorta (vDA) contain cells with a HSC phenotype.^15^ Altogether these findings suggest that HSCs directly originate from hemogenic endothelial cells. More recently, hemogenic endothelium (HE) cells have been shown to also give rise to EMPs generated in the yolk sac,^3^ a process that is recapitulated *in vitro* in ES cell culture systems.^22,27,28^ In contrast to AGM derived HE, yolk sac HE expresses the hematopoietic marker c-KIT.^3^ Even reprogramming of fibroblasts to blood cells was shown to proceed through a HE intermediate.^29-31^ Together, these recent results highlight the pivotal role of the HE cell population and the EHT process in the *de novo* generation of blood cells.

Although HE has now been clearly established as the cellular source of the first blood cells *in vivo* and *in vitro*, the molecular and cellular mechanisms orchestrating this intriguing trans-differentiation remain largely unknown. The EHT process is characterized by the loss of endothelial identity concomitant with the acquisition of a round, non-adherent, cellular morphology and the gain of hematopoietic cell surface marker expression. One important clue in understanding this process was provided by the observation that the transcription factor RUNX1 is critical for the generation of definitive blood cells by EHT.^17,21,32-35^ In the absence of this transcription factor, HE cells do not lose their endothelial identity nor do they acquire a hematopoietic fate. Instead, the cells remain mostly adherent, associated in tight clusters and the few cells that separate from HE clusters rapidly die through apoptosis.^21,36^ Taking advantage of this critical role of RUNX1 in the EHT process, we identified through genome-wide gene expression studies the transcriptional repressors GFI1 and GFI1B as direct transcriptional targets of RUNX1 during the EHT.^37^ These 2 homologous nuclear zinc finger proteins share a C-terminal domain containing 6 C_2_-H_2_-type zing finger motifs mediating their DNA binding activity and a N-terminal SNAIL/GFI-1 (SNAG) domain required for their repressive activity.^38,39^ GFI1 and GF1B have already been implicated in the adult hematopoietic system.^39,40^ *Gfi1* is expressed in HSCs, granulocyte-macrophage progenitors, B cells, granulocytes and immature T lymphocytes ^41-44^ whereas *Gﬁ1b* is highly expressed in HSCs, erythroid and megakaryocytic cells.^45^ To investigate the relevance of these 2 proteins in the EHT, we evaluated their ability to rescue this transition in *Runx1*^−/−^ HE cells. We observed that ectopic expression of *Gfi1*, or *Gfi1b*, restored many features of the EHT process. The cells expressing either GFI1 proteins acquired both a round, non-adherent morphology and the expression of early hematopoietic markers, while losing the expression of endothelial genes. However, these newly generated round cells were not able to generate hematopoietic colonies, indicating that the rescue of the EHT was incomplete. To confirm the association of GFI1 and GFI1B with the EHT *in vivo*, we analyzed in detail their expression in the mouse AGM region.^46^ We observed that during the EHT process, *Gfi1* is specifically expressed within the dorsal aorta in endothelial cells and cells within emerging IAHC, whereas *Gfi1b* expression was more associated with the fully formed IAHC. Furthermore, transplantation of the E11.5 AGM endothelial cells expressing *Gfi1* and/or *Gfi1b* resulted in long-term repopulation of irradiated recipient mice directly demonstrating that HSC potential at E11.5 resides within the GFI1(s) expressing endothelial cell compartment. These results indicate that the expression of *Gfi1* in endothelial cells readily distinguishes HE from normal, non-hemogenic endothelial cells and that GFI1 could be an important effector of RUNX1 function in the EHT process. Interestingly, we also found that in the yolk sac, *Gfi1* expression was associated with FLK1^+^ or CD31^+^ endothelial cells at sites of EMPs emergence (Fig. 1). In contrast, GFI1B was mostly found in cells negative for endothelial markers. *Gfi1* expression in yolk sac endothelium also coincided with the expression of c-KIT, a marker of hemogenic endothelial cells in the yolk sac,^3^ but not in the AGM where its expression marks subsequent hematopoietic clusters.^46^ The observation that GFI1 concurs with c-KIT and endothelial markers expression, and therefore potential hemogenic endothelium, suggested that GFI1 could also be critical for the extra-embryonic EHT.

**Figure 1.**
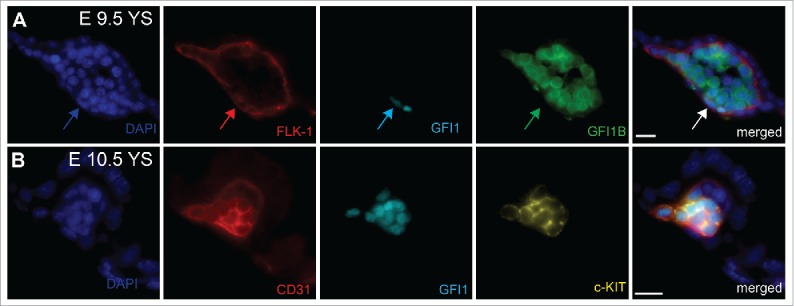
Immunostaining on E9.5 and E10.5 Yolk sacs (A) Arrows indicate the expression of GFI1 in flat FLK-1^+^ endothelial cells in E9.5 yolk sac. GFI1B is detected in intravascular round cells. (B) Co-expression of GFI1 and c-KIT in CD31^+^ E10.5 hemogenic endothelial cells. YS = Yolk Sacs. Scale bar = 10μm.

Although these previous findings strongly suggested the importance of GFI1 and GFI1B in the EHT, none of their respective knockout recapitulated the early block in EHT and the embryonic lethality observed at E12.5 in the absence of RUNX1.^47,48^ GFI1 deficiency is not embryonic lethal and results mainly in deafness, neutropenia and reduction in HSC self-renewal capacity,^41,43,44,49,50^ whereas *Gfi1b* knockout leads to embryonic lethality at E14.5 due to a deficiency in erythroid and megakaryocyte development.^51^ We hypothesized that the lack of an early phenotype might be due to a functional compensation for the loss of one gene by the other. The two GFI1 and GFI1B proteins exhibit very high level of homology in their functional domains and were previously shown to be functionally interchangeable in the adult hematopoietic system.^52^ In addition, both proteins auto-regulate themselves and cross-regulate each other.^53-57^ In line with a possible functional compensation, we observed the up-regulation of *Gfi1b* expression in *Gfi1* deficient AGM HE cells ^46^ although *Gfi1b* is not normally expressed in these HE cells in wild type embryos. To therefore evaluate the functional relevance of GFI1 and GFI1B in EHT, we examined the consequences of deleting both proteins during embryonic development using *Gfi1* and *Gfi1b* GFP knock-in mice. We first observed that deficiency in both proteins resulted in an earlier lethality than either single deficiency, further supporting the hypothesis of a functional compensation between these 2 highly homologous proteins. In the double knockout embryos, strong defects in the EHT were also observed; GFP^+^ blood cells normally generated from the yolk sac in heterozygous animals were absent from the circulation in the double knockout animals. Furthermore, IAHC were not observed in the AGM. Instead, we found GFP^+^ cells accumulating in the yolk sac or embedded within the endothelial lining of the dorsal aorta. Interestingly when these yolk sac GFP^+^ cells from the double knockout embryos were isolated and replated, they readily generated hematopoietic colonies. These results indicate that although the GFP^+^ cells were not disseminated in the circulation they had already committed to a hematopoietic cell fate. In contrast, the GFP^+^ endothelial cells present in the dorsal aorta did not generate any hematopoietic colonies following either direct replating or after a maturation step by co-culture on OP9 cells. These findings suggest that blood commitment can take place in absence of both GFI1 and GFI1B in the yolk sac but not in the AGM. Alternatively, committed blood cells are generated in the AGM in the absence of both GFI1 proteins, but these hematopoietic cells, such as HSCs, might be more dependent on the presence of at least one of the GFI1 protein. Supporting this hypothesis, the conditional deletion of *Gfi1b* in the bone marrow of adult *Gfi1* deficient animals, generating double knockout cells, result in complete loss of HSCs indicating that either GFI1 or GFI1B are required to maintain HSC *in vivo*.^50,58^

Although these data demonstrated the critical requirement for GFI1 and GFI1B in the EHT, the molecular mechanism associated with their function in this process still remained unknown. GFI1 and GFI1B have been shown to repress transcription in MEL (murine erythroleukemia) cell line, by recruiting the chromatin regulatory CoREST complex.^59,60^ The CoREST complex includes the histone demethylase LSD1 (KDM1A) and the histone deacetylases, HDAC1 and HDAC2.^61^ To investigate if this complex was involved in EHT, we examined the consequences of pharmacological LSD1 inactivation on this transition during the *in vitro* differentiation of ES cells. LSD1 inhibition impaired the emergence of round non-adherent cells in the supernatant of those cultures, affected the acquisition of early hematopoietic markers and perturbed the loss of endothelial markers. A similar phenotype was obtained upon the genetic deletion of *Lsd1* in HE cells generated from ESCs carrying a tamoxifen-inducible conditional *Lsd1* knockout.^62^

In order to identify the genome-wide transcriptional changes induced by GFI1 and GFI1B through the recruitment of LSD1 during EHT, we compared global gene expression profiles upon LSD1 inhibition. We found that genes implicated in the development of the cardiovascular system were expressed at higher levels in LSD1-inhibited cells than in control cells. Conversely, transcripts associated with hematological system development/function and cell morphology were found at lower levels. We also mapped GFI1 and GFI1B binding sites in HE cells using the DamID (DNA adenine methyltransferase Identification) strategy. This alternative approach to chromatin immune-precipitation relies on the deposition of “methylation tags” around the binding sites of the Dam-fused transcription factor under investigation by the *E.coli* DNA adenine methyltransferase (Dam).^63,64^ We cross-compared the lists of genes bound by GFI1 and/or GFI1B with the list of genes differentially expressed when LSD1 activity is blocked to identify direct transcriptional targets. The resulting list of candidates contained several genes with well-established role in stemness (*Lgr5, Lin28A, Sall1*), as well as genes involved in cardiovascular development, blood vessels maintenance and remodelling. Interestingly, many of these genes were also previously shown to be bound by RUNX1 during EHT, or to contain RBPJ binding sites, suggesting that RUNX1, NOTCH and GFI1(s) might be participating together in the regulation of these genes.

Altogether, these studies suggest a model of regulation of the EHT process where RUNX1 is first expressed in HE and induces the expression of *Gfi1* and *Gfi1b*. These two proteins bind to genes associated with the global maintenance of endothelium identity, and cellular adhesion, and recruit the CoREST complex to epigenetically silence the endothelial program (Fig. 2). This leads to the acquisition of a round non-adherent cellular morphology, allowing the release of newly formed blood cells into the circulation. The precise role of NOTCH signaling in the context of RUNX1 is still unclear but is comprehensively investigated by other laboratories.^65-67^ It is interesting to note here that GFI1, and GFI1B, have also been respectively shown to be important for the reprogramming of endothelial cells to blood cells^68^ or of fibroblasts to hemogenic endothelium.^30^ We also recurrently found RUNX1 binding sites with GFI1 and GFI1B binding sites,^46,69^ suggesting a model in which RUNX1 and GFI1(s) interact and cooperate to shut down the endothelial program. Indeed, RUNX1 and GFI1 were shown to be part of common transcriptional complexes in hematopoietic progenitors^70^ and we observed that these proteins can immuno-precipitate each other (unpublished data). We therefore propose that RUNX1 first binds to endothelial genes and increases the expression of GFI1(s) repressors that along with LSD1 actively induce the epigenetic silencing of the endothelial program (Fig. 2).

**Figure 2.**
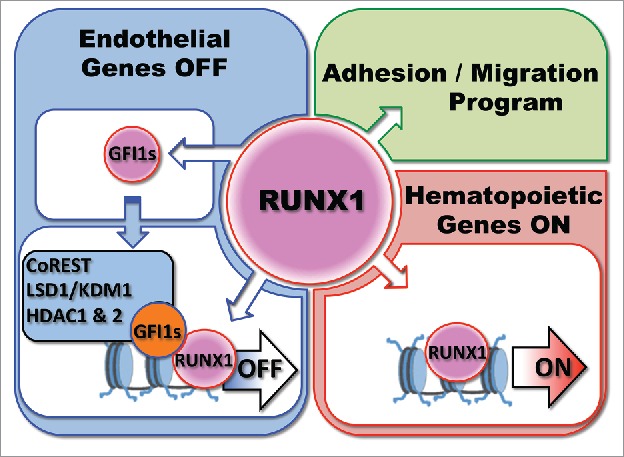
Model of Regulation by RUNX1 and GFI1(s) of the Endothelial to Hematopoietic Transition. The model is discussed in the text.

The observation that the expression of *Gfi1*, or *Gfi1b*, in *Runx1*^−/−^ deficient HE cells induces the loss of endothelial identity but does not confer hematopoietic potential suggest that RUNX1 also activates the expression of a different set of genes required for blood commitment (Fig. 2). One possibility is that the acquisition of a blood cell fate proceeds through a global activation of the expression of hematopoietic genes. Indeed we found in collaboration with the Bonifer laboratory that in the absence of RUNX1, many regulatory genes such as *Scl/Tal1, Fli1, Lmo2* and *Cebp*β are already expressed and many hematopoietic genes are bound by C/EBPβ, SCL/TAL1 and FLI1.^69,71^ RUNX1 expression causes a rapid shift in the binding pattern of these transcription factors toward that observed in hematopoietic precursor cells in the absence of overt precursor formation. Moreover, RUNX1 initiates the formation of new transcription factor complexes with a concomitant increase in histone acetylation at a large number of newly formed cis regulatory elements. These data suggest that the acquisition of hematopoietic cell fate might result from a global reorganization of lineage-specific transcription factor assembly controlled by RUNX1 rather than the induced expression of a few critical genes. Nonetheless the exact mechanisms by which RUNX1 promotes hematopoietic cell fate, the stage at which this is initiated, as well as the complete inventory and function of key downstream target genes, remains to be uncovered.

Intriguingly, although we previously found that *Gfi1* is among the earliest target genes bound and up-regulated by RUNX1b, the isoform of RUNX1 expressed in HE, the ontology of RUNX1b regulated genes in early HE revealed an upregulation of the expression of genes involved in angiogenesis, cell adhesion and migration.^63,72^ This suggests that at the initial stage of hematopoietic development, RUNX1b first organizes the formation of HE clusters that is required for the release of blood progenitors. Outside of the hematopoietic context, this endothelial-epithelial RUNX1 transcriptional signature might also reflect the recently uncovered role of RUNX1 in epithelial-based tumor formation and progression. In particular RUNX1 targets that associate with cell migration in HE may represent important regulators of the potential metastatic role of RUNX1 in solid tumors. It is therefore possible that RUNX1 performs 2 seemingly opposite functions in HE, first binding and activating the expression of genes involved in a cell adhesion program followed by the recruitment of GFI1(s) to silence the endothelial identity of HE.

In conclusion, our recent studies provided exciting new insights into the molecular mechanisms driving the EHT and revealed the critical roles of GFI1(s) in this process. These findings extend previous studies that have identified critical transcription factors for the upstream cell fate choice leading to the development of the HE from mesodermal/hemangioblast progenitors^27,73,74^ or implicated in the EHT process. These include ETS factors,^75-77^ in particular ETV2,^78-82^ and other transcription factors such as SOX7,^83-86^ SCL/TAL1,^21,87-90^ HOXA3,^91^ SOX17,^92-94^ GATA2 ^95,96^ and FOXF1.^97^ The next challenge will be to determine how the combination of these transcription factors and the epigenetic machinery together dynamically orchestrate the gene regulatory networks that drive the generation of blood cells.^74^ Along this line, our finding that LSD1 is critical for the EHT is starting to unravel how this process is regulated at the epigenetic level. In addition, it will be important to identify the components of the cellular niches that trigger and support the generation of the different types of blood cells by EHT in order to reproduce these processes *in vitro* to produce cell populations appropriate for clinical purposes.^98-103^ Along this line, we recently demonstrated the relevance of a specific combination of cytokines,^104^ heparin sulfates^105,106^ and graphene oxide^107^ in supporting the development of blood cells *in vitro*. Altogether the *Gfi1* GFP knockin model represents a powerful tool to address in the future these outstanding questions and study the EHT process in more detail as the expression of *Gfi1* in the endothelium can be used to accurately identify and purify hemogenic endothelium and cells undergoing EHT. This should not only allow a better characterization of the molecular program that underpins blood cell emergence but should also lead to the identification of the specific molecular and cellular mechanisms that control the generation of different lineages during the successive waves of blood generation.
